# High incidence of metastatic disease in primary high grade and large extremity soft tissue sarcomas treated without chemotherapy

**DOI:** 10.1186/1471-2407-6-160

**Published:** 2006-06-18

**Authors:** Benedikt Leidinger, Thomas Heyse, Andreas Schuck, Horst Buerger, Philipp Mommsen, Thomas Bruening, Susanne Fuchs, Georg Gosheger

**Affiliations:** 1 Philipps-University Marburg, Department of Orthopaedics and Rheumatology, 35043 Marburg, Germany; 2 University Hospital Muenster, Department of Radiation Therapy, 48149 Muenster, Germany; 3 Institute of Pathology, University of Muenster, 48149 Muenster, Germany; 4 University Hospital Muenster, Department of Orthopaedic Surgery, 48149 Muenster, Germany

## Abstract

**Background:**

The risk of metastasis and the survival in patients with primary extremity soft tissue sarcomas is worse when tumour size is large and the grade of malignancy is high. Such tumours may receive chemotherapy and/or radiation therapy (RTX) for optimising local control. Irradiation can either be applied preoperatively or after tumour resection. The question arises if the kind of RTX in the absence of chemotherapy influences the outcome concerning local control, metastatic disease, survival and complications.

**Methods:**

We retrospectively reviewed the clinical outcome of 233 patients with a primary extremity soft tissue sarcoma treated between 1990 – 2000 with a mean follow-up of 35.8 (4–120) months in our institute. 41 patients had high grade, deep and large tumours (>8 cm), an AJCC stage III (no evidence of metastasis prior to treatment) and were treated with limb salvage surgery and irradiation but stayed without additional chemotherapy. Two groups of patients were compared: the first group received postoperative RTX after tumour resection (n = 33); the second group was treated with preoperative RTX (n = 8). Both groups did not differ concerning clinical parameters. We analysed primary and secondary outcomes.

**Results:**

56% (23/41) of the population developed metastatic disease, 24% (10/41) local recurrence. The risk of metastasis was higher in the group with preoperative irradiation (p = 0.046). The overall (p = 0.0248) and relapse free survival (p = 0.104) were worse in this group. The delay to tumour resection amounted 8 weeks on average in the preoperative group. Local control was not different (p = 0.38) in both study groups. Wound infections and other combined therapy related complications were equally distributed (p = 0.22).

**Conclusion:**

Without chemotherapy there remains a high risk of metastasis in AJCC grade 3 patients. In high risk patients treated without chemotherapy the elapsed time to tumour resection after preoperative radiation might contribute to the development of metastasis. This outcome may support the thesis that a combination of RTX and offensive multimodal treatment protocols is advantageous in such a subset of patients

## Background

Since chemotherapy unlike in bone sarcomas is considered less effective in soft tissue sarcomas, wide resection and postoperative RTX with 60–66 Gy for optimizing local control is the standard treatment for such high grade tumours [1-7]. Irradiation can be administered before, during or after excision of the primary tumour with entirely acceptable local control rates of 70–100% [4,6,8-20]. The advancement of when to apply irradiation still remains a controversy [7,21-26]. Furthermore, the impact of local recurrence on overall survival is still not clear [4]. Some centres use irradiation for preoperative down staging of the tumour making limb salvage procedures applicable [23] or for reduction of the radiation dose and field size [27-29].

Development of metastasis is the most limiting event in sarcomas [21,23,26,30,31] and is dependent on tumour size and grade [13]. Heise et al. [32] reported a risk of 65% for metastasis in high grade and large extremity soft tissue sarcomas. Local recurrence is unlikely a major source of metastasis [33]. With this in mind, this study focuses on a group of patients with high grade, deep and large extremity soft tissue sarcomas which leveled identical values in all clinical parameters but differed only in the way when RTX was applied, either preoperative or postoperative. It was analyzed if one therapy is advantageous in such a population.

## Methods

Between 1990 and 2000 a total of 233 patients were treated for an extremity soft tissue sarcoma at the University Hospital Muenster, Department of Orthopaedic Surgery. Data regarding tumour stage, grade, site, size, and depth, time to treatment initialisation, surgical margin, radiotherapy, local control and relapse-free respectively overall survival were evaluated by review of the hospital and office records. If this was not possible direct contact with the referring physicians or patients was established. Follow-up data was successfully obtained on all patients. Patients were excluded from this analysis for the following reasons: primary metastasized tumour (AJCC stage IV), secondary malignancy, low grade, superficial or small tumour (<8 cm), patients with chemotherapy or prior radiotherapy or patients without primary limb salvage surgery. Furthermore, all rhabdomyosarcomas were excluded due to different treatment protocols. The study protocol was presented to the local ethics committee. Since we exclusively dealt with retrospectively and anonymously acquired data no official approval was necessary.

Using these criteria we reviewed 41 patients aged 18–77 (48 ± 17) years with a primary, nonmetastatic, high grade, deep and large soft tissue sarcoma of the extremities who received limb salvage surgery and RTX either preoperatively or postoperatively. The average length of follow-up was 35.8 (4–120) months. All included patients were staged no longer than 4 weeks prior to biopsy showing no evidence of metastatic disease. The elapsed time from biopsy to initialisation of therapy averaged 8.5 days in the postoperative group and 7.8 days in the preoperative group (p = 0.42, Mann-Whitney-U).

Since 1990 the principles of surgical management were standardized using anatomical terms of compartment surgery as described by Enneking et al. [34]. All surgical resections were performed by a musculoskeletal oncologic surgeon.

Applying radiotherapy preoperatively or postoperatively was decided individually after review of all imaging studies and interdisciplinary discussion with the involved colleagues and patients.

In this analysis, two patient groups were retrospectively studied. The first group was treated with postoperative RTX after tumour resection. The second group received preoperative irradiation. The distribution of these 41 sarcomas concerning histological type, anatomic site, tumour size, age, sex, resection margin and stage is presented in Table 1 and Table 2. The most underlying disease was malignant fibrous histiocytoma (n = 18) in 44% of all cases.

The mean total radiation dose administered to all 41 patients amounted to 56.1 Gy. The mean preoperative radiation dose was 50 (48–51) Gy applied over a mean duration of 35 days. Preoperative irradiation was initialized 5–11 days after biopsy. The planning target volume for preoperative radiotherapy included the primary tumour with a safety margin of usually 5–7 cm in longitudinal and 2 cm in axial direction. The elapsed time from initialisation of preoperative RTX to tumour resection was delayed by 8 (7 to 9) weeks on average compared with the postoperative radiation group.

Tumour resection in the postoperative group followed 5–14 days after biopsy. The mean postoperative radiation dose was 60 (58–66) Gy. 1.8–2.0 Gy were applied 5 days a week for a total of 6–8 weeks. Postoperative radiotherapy was begun after completion of primary wound healing no later than 4 weeks after tumour resection. It was delayed in case of marked cutaneous reactions and wound complications. The planning target volume for postoperative radiotherapy included the primary tumour volume with a safety margin of 5–7 cm in longitudinal and 2 cm in axial direction including scars and drain site for up to 50 Gy and a boost of 10–16 Gy to the initial tumour volume and an additional margin of 2 cm. Circumferential limb irradiation and unnecessary irradiation of joints and healthy tissue was avoided.

The resected tumour specimen was histologically analysed by the Gerhard-Domagk-Institute of Pathology at the University Hospital Muenster for final diagnosis as to histopathological type, grade and resection margin. Grading was based on degree of tumour differentiation, mitoses and necrosis [35]. Pathological evaluation was used to determine tumour size and the amount of tumour necrosis. The extent of histological necrosis in the resected tumour was graded according to the criteria of Willet et al. [36]: Grade 1: <50% necrosis, Grade 2: 50–80% necrosis, Grade 3: >80% necrosis. On average one block per cm of the largest tumour diameter was evaluated by routine histology (H&E stain). The amount of tumour necrosis was described in percentage of the evaluated tumour area.

Complications of the combined therapy (irradiation and surgery) were evaluated (Table 3). Complications were defined as operatively treated delayed wound healing, persistent lymph edema or pathological nonmetastatic fracture of the radiated extremity without prior partial bone resection.

All sarcomas were staged using the American Joint Committee on Cancer (AJCC) staging system. Standard software (SPSS for Windows, version 11.5.1) was used for statistical analysis. In order to determine patient survival Kaplan-Meier curves were plotted. Survival estimates were performed for overall survival, relapse-free survival, local and distant control using mean and standard deviation with 95% confidence intervals (CI). The log rank test was used to compare Kaplan-Meier curves. Additionally, the chi square test and Mann-Whitney-U test were used in order to evaluate the association between two variables.

## Results

### I. Overall survival

**Figure 1:**
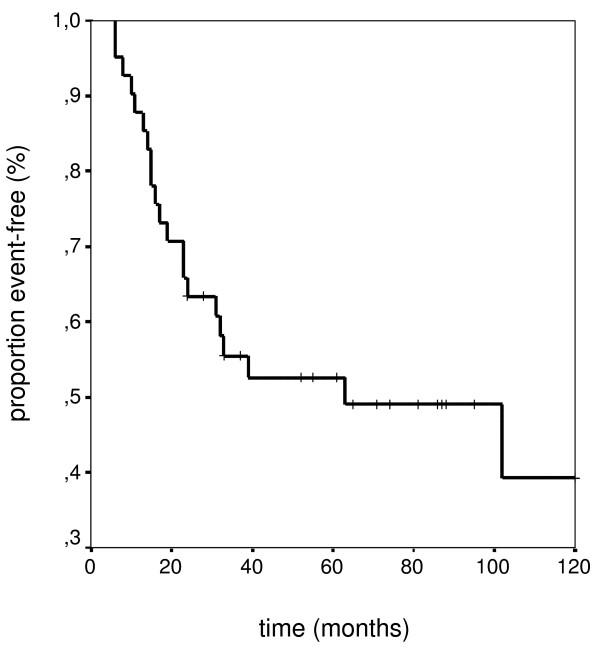
Overall survival of the entire collective (n = 41).

**Figure 2:**
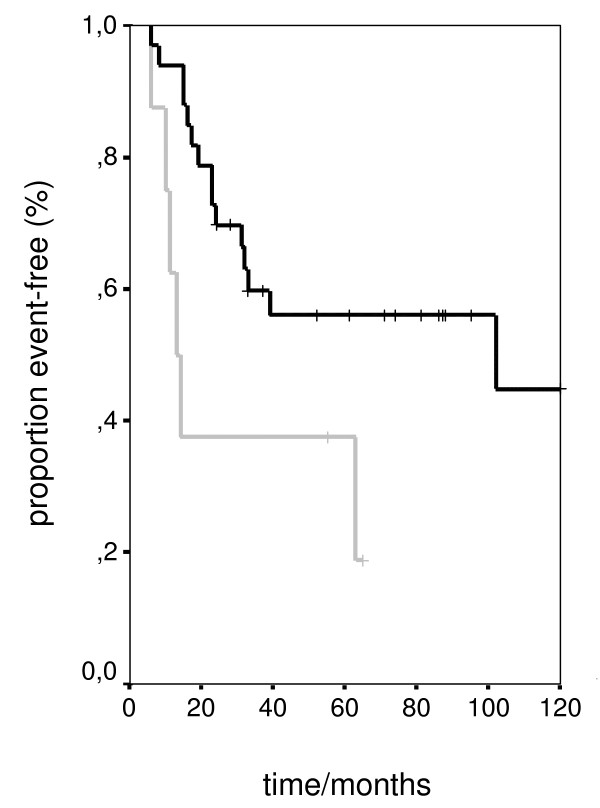
Overall survival for patients with preoperative (grey line) versus postoperative radiotherapy (black line).

The overall survival of the entire study group (n = 41) was 52.6% after 5 years and 39.3 % after 10 years (Fig. 1). The mean survival was 68 months (s ± 8, 95% CI 53–84). The Kaplan-Meier-analysis (Fig. 2) showed a significant difference in overall survival between the patient group who had received radiotherapy preoperatively and the group who was treated with postoperative irradiation (p = 0.0248, log rank) favoring the postoperative group. At the 2.5-year time point, overall survival for patients receiving preoperative radiotherapy was 37.5% (s ± 17) versus 69.7% (s ± 8) for the postoperative radiation group and at the 5-year time point 18.8% (s ± 16) versus 56.0% (s ± 9). The average survival time of these patients who were treated with postoperative radiotherapy amounted to 75 months (s ± 9, 95% CI 58–92). In contrast, the patients who underwent preoperative irradiation survived 31 months (s ± 9, 95% CI 13–49) on average.

### II. Relapse free survival (RFS)

**Figure 3:**
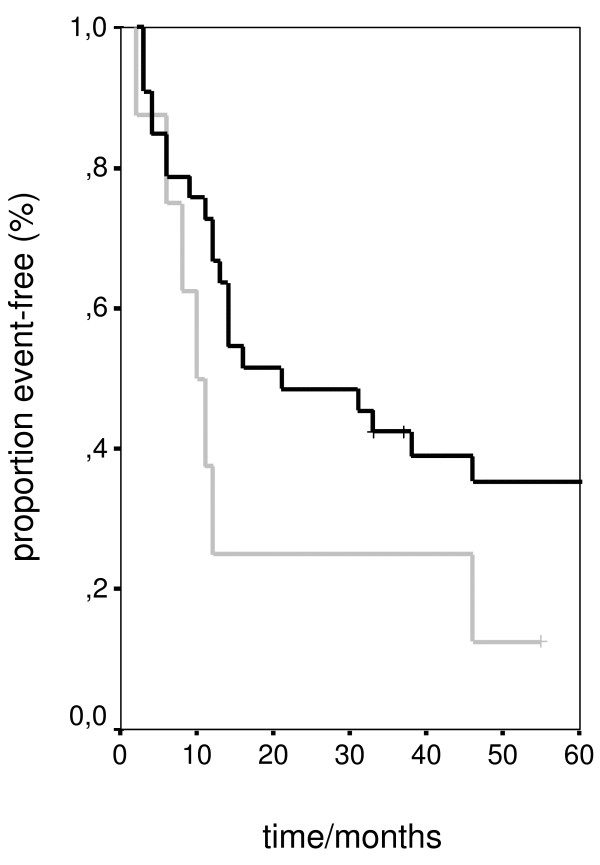
Relapse-free survival for patients with preoperative (grey line) versus postoperative radiotherapy (black line).

In the preoperative radiation group 7 of 8 patients (87.5%) developed a disease relapse, 6 during the first year after tumour resection (metastasis), one patient after 46 months (metastasis), one patient developed metastasis after 2 months and local recurrence after 3 months. Among the patients receiving postoperative radiation the number of disease relapses was 22 (66.6%). The RFS for patients with preoperative irradiation was 25% (s ± 15) at 2.5-years time point versus 48.5% (s ± 9) for the postoperative radiation group. On average, patients who received irradiation after tumour resection survived 50 months (s ± 9, 95% CI 33–67) in complete remission compared with 19 months (s ± 7, 95% CI 6–32) in the preoperative radiation group (Fig. 3). Despite this trend a significant difference between both patient groups concerning relapse-free survival could not be demonstrated (p = 0.104, log rank).

### III. Metastasis

Overall, 23 patients (56.1%) developed distant metastases in both patient groups, 18 (43.9%) remained free of any metastases. The number of metastases in the preoperative radiation group was 7 (87.5%) and in the postoperative group 16 (49.5%). In the following square table (Table 4) a significant higher risk of metastases could be demonstrated for patients who had received preoperative irradiation (p = 0.046, chi square). The average metastasis time (time from diagnosis to metastasis) for both studied patient groups amounted to 15.61 (3–64) months. In the preoperative group 6 patients suffered metastatic disease in the first year after tumour resection (2, 5, 5, 6, 8 and 12 months) and one after 46 months. On average, patients with preoperative radiotherapy developed distant metastasis about 5 months earlier than patients who had undergone irradiation after tumour resection: the average metastases time was 12 (5–46) months with preoperative versus 17 (3–64) months with postoperative irradiation (p = 0.72, Mann-Whitney-U). Margin status (p = 0.68, chi square) and local recurrence (p = 0.24, chi square) did not influence the development of metastatic disease. The clinical features of preoperative and postoperative treated patients who developed distant metastasis are displayed in Tables 5 and 6.

### IV. Local recurrence and local recurrence free survival

The total number of local recurrences was 10 (24.4%). In 9 of 33 patients (30%) who underwent postoperative radiotherapy local recurrence was diagnosed after an average time (from diagnosis to local recurrence) of 18 (6–46) months. Patients with preoperative radiotherapy had equal local control (p = 0.38, chi square). Only one patient (12.5 %) developed local recurrence after 3 months respectively (Table 7). The overall average local recurrence time was 16 (3–46) months. The margin status of either marginal or wide resection did not influence the development of local recurrence, 7/22 recurrences with marginal, 3/19 recurrences with wide resection (p = 0.23, chi square). The metastasis rate was independent of the local control status of each group (p = 0.24, chi square). The local control status did not influence the overall survival (p = 0.45, log rank). Patients with a local recurrence had a mean survival of 77 (s ± 14, 95% CI 49–104) months and patients without a local recurrence had a mean survival of 66 (s ± 9, 95% CI 48–83) months. Actuarial analysis of the local recurrence-free survival of patients did not reveal a significant difference between the preoperative and postoperative group: the proportion of patients free of local recurrence after 5-years for the preoperative radiation group was 87.5% versus 77.5% for the postoperative patients (p = 0.85, log rank). The mean local recurrence free survival was 91 (s ±, 95% CI 76–105) months in the postoperative group and 58 (s ± 7, 95% CI 44–71) months after preoperative radiation. Local relapse was treated by resection with limb sparing surgery (6) or salvage amputation (2) and in two cases of multiple metastases palliatively.

The amount of tumour necrosis in cases with by preoperative irradiation was less than 50% in 5/8 patients and 50–80% in the other 3/8 cases. There was no correlation between amount of necrosis and the development of metastasis or local recurrence. However, a definite distinction between original and radiation induced tumour necrosis was not possible since in most cases only a small biopsy for the establishment of the tumour diagnosis could be compared with the final resection specimen.

## Discussion

Although RTX can cause transient or permanent loss of lower limb function [4,24] it is regarded to improve local control of high grade soft tissue sarcomas. This enables the surgeon to perform limb sparing surgery with the same results concerning local tumour control as with radical resection or amputation. Compared with a 50–70% relapse free survival of nonirradiated patients [4,8,19,37] the success rate after RTX (70–100%) is much higher [3,6,7,12-15,17,18,28,31,38]. Whether to apply irradiation preoperatively or after tumour resection has been matter of debate in previous studies [7,21,24,25].

Preoperative radiation may be indicated if tumour size is very big (>10 cm), if the localization is close to vital structures and radical resection otherwise is needed [11,13,21-23,39,40]. The histological response to preoperative radiotherapy has been more favorable in large and high grade tumours [13,21,36].

Preoperative radiation may still be useful for patients with tumours not amendable to a limb salvage procedure due to proximity of neurovascular structures [22,40]. In these cases, brachytherapy can also be an alternative [16]. Preoperative radiation has been reported to be beneficial in tumours after a shell out procedure resulting in an intralesional margin [22].

The theoretical advantages of preoperative irradiation include decreased intraoperative seeding of viable tumour cells in the operative field, sterilization of lymph node metastasis outside the operative field, a smaller tumour volume due to necrosis with formation of a pseudo capsule facilitating surgical resection [22,23,29,36] and a reduced toxicity with smaller median radiation dose and field size [27] which is important since Yang et al. (1998) reported about transient lower limb strength and decreased range of motion in patients after postoperative radiation with 63 Gy on average. Also, preoperative irradiation seems to offer lesser late radiation morbidity by diminished fibrosis, joint stiffness and edema [41].

Disadvantages are an increased number of wound infections [7,11,13,22,25,42,43] with a possible detrimental effect on patient function [24] and other complications [2,13,44].

Concerning local control a positive outcome of preoperative administered irradiation is reported by Barkley et al. (1988) [11], Brant et al. (1990) [39] and Sadoski et al. (1993) [28]. Suit et al. [13] compared preoperative versus postoperative radiation for soft tissue tumours and found no difference in overall survival but local control of large tumours was improved by preoperative irradiation. Suit and Spiro (1994) [19] reported a local success rate of 100% and 79% of 15 cm and 20 cm large tumours after preoperative radiation whereas a rate of only 50% and 67% was achieved with postoperative irradiation. Cheng et al. (1996) [22] compared preoperative with postoperative RTX in similar patient groups and did not find significant differences concerning local control and survival. More decisive for local control than the kind of irradiation is a correct tumour resection [28]. A positive resection margin deteriorates local control significantly [28, 45]. Karakousis and Zografos (2002) [46] and Karakousis et al. (1986) [1] treated tumours with wide resection margins with surgery alone and tumours with a margin <2 cm with additional irradiation of 45–60 Gy. The 5-year local recurrence rate was 7–19% and 17–24%, respectively. This displays the potential of RTX to improve local control in problematic cases. The difference in local failure between wide and marginal resection with and without radiation concerning to Alho et al. (1989) [47] was 8% to 10 or 37%. Marginal resection alone has a convincing high risk and increased local failure rate [45]. Our local control rate in the two observed groups was not statistically different (p = 0.38). The resection margins were equally distributed in both groups. Local relapse was treated by resection (six cases) or salvage amputation (two cases) and palliatively in two cases of multiple metastases.

Local control status has moderate influence on the development of metastasis [33, 45, 48, 49]. Metastatic disease is the most serious and limiting event in sarcomas [21,23,26,29,30,32]. Suit et al (1988) [13] reported a 60% risk of metastatic disease in 15–20 cm large extremity soft tissue sarcomas. In our study group (n = 41) with comparable tumour size 56% of the patients developed distant metastasis although preoperative staging in all patients no later than 4 weeks prior to treatment was negative. The preoperative radiotherapy group was associated with 7/8 (87.5%) and the postoperative group with 16/33 (49.5%) cases of metastatic disease (p= 0.046, chi square). Occurrence of metastasis was not influenced by surgical margin (p = 0.68) or local control status (p = 0.24). The high percentage of metastatic disease caused a nonsignificant worse relapse free survival of the preoperative radiation group (p= 0.104). A similar outcome has earlier been reported by Cheng et al. (1996) [22] also as a tendency in a restricted group of their patients favouring the relapse free survival of the postoperative radiation group. Virkus et al. (2002) [23] reported 48% metastasis of their preoperative group and a local control rate of 86%. Wanebo et al. (1995) [30] reported 98.5% local control rate but 38% metastases after preoperative radiation despite simultaneous doxorubicin based chemotherapy. Current prospective trials use chemotherapy to improve distant disease-free survival resulting in a significant improvement of 75% versus 44% compared to a historical control group [26]. The combination of chemotherapy with regional hyperthermia has shown promising results [50].

In a prospective randomised study of preoperative versus postoperative RTX by O'Sullivan et al. (2002) [25] regional and distal failure rates were identical but in this study low grade (17%) and superficial (16–21%) tumours were included. Nevertheless, this strong study suggests that the results of our retrospective study are not universal valid. An acknowledged deficiency of our study might be the preselection bias inherent in all review studies making validity and reliability of the results questionable. Although we did not intend to preselect patients for any of the two examined groups our patients are not equally proported (33-8) and smaller than other reported series. Despite, they are restricted to a well-defined cohort of patients treated in a uniform manner and relatively well balanced (see Table 1, 2). Average age, stage, tumour size, depth, anatomic localisation and the resection margin were equal in both groups. In addition, our study group quite apparently displays an antiquated therapy regime for in this group despite a high risk tumour no chemotherapy was used. Chemotherapy today is a widely accepted and important part of all randomised evidence-based treatment protocols. We decided to enrol only patients who did not receive chemotherapy to exclude any possible influence of this treatment on survival and the incidence of metastasis in order to have a unique statement about the possible influence of radiotherapy.

An average loss of time from initialization of preoperative irradiation to tumour resection of 5 weeks and additional 3 weeks to allow the soft tissue to recover [23] can be expected. The loss of time to surgical resection with viable tumour in situ when irradiation is not fully effective might deteriorate distant disease control. Willett et al. (1987) [36] could find >80% necrosis in 69% of his patients with large tumours >10 cm after preoperative radiation. Robinson et al. (1992) [40] showed in 60% of their preoperative radiated patients a response with tumour reduction but without clinical correlation. Other experimental and radiographic studies report that a reduction in tumour volume may only be achieved in 40–60% with even tumour progression in 12–15% [39]. Pitson et al. (2004) [51] stated that preoperative radiation reduced tumour volume (-59%) in liposarcoma but was ineffective in diminishing tumour size in other entities (MFH, +7% volume). Hew et al. (1994) [52] demonstrated only 26% of other than liposarcoma tumours with >80% necrosis. Our study group had mainly <50% tumour necrosis after preoperative RTX unrelated to further clinical outcome. The value of tumour necrosis estimation for radiosensivity and the clinical course of different entities remains arguable [53]. Another problem is that negative staging does not rule out occult micro metastasis. This might bias our findings concerning metastasis after radiotherapy. At least, this problem affects both study groups.

## Conclusion

Without chemotherapy there remains a high risk of metastasis in AJCC grade 3 extremity soft tissue sarcoma patients. Pre- or postoperative radiation seems to offer equal local control. When no chemotherapy is applied the elapsed time to tumour resection after preoperative radiation might contribute to the development of metastasis especially when the response to therapy is low. This outcome may support the thesis that a combination of RTX and offensive multimodal treatment protocols is advantageous in such high risk patients.

## Abbreviations

Abbreviations found in the text are defined where used first.

## Competing interests

The author(s) declare that they have no competing interests.

## Authors' contributions

BL: Conception and design; analysis and interpretation of data. TH: analysis and interpretation of data, drafting and revision of the manuscript. AS: Acquisition of data. HB: acquisition of data, analysis and interpretation of data. PM: acquisition of data. TB: Acquisition of data. SF: contributions to conception and design, revision and final approval of manuscript. GG: conception and design. All authors read and approved the final manuscript.

## Funding

All funding for the study and for the manuscript preparation was taken over by the Department of Orthopaedics and Rheumatology, Philipps-University Marburg and the Department of Orthopaedic Surgery, University-Hospital Münster.

## Pre-publication history

The pre-publication history for this paper can be accessed here:



## Figures and Tables

**Table 1:** Summary of patients' diagnoses

**diagnosis**	**patients**
malignant fibrous histiocytoma	18
leiomyosarcoma	8
liposarcoma	7
neurogenous sarcoma	4
synovial sarcoma	4
**total:**	**41**

**Table 2:** Summary of study group patient data

	**Radiotherapy**	
	**preoperative**	**Postoperative**

cases	8	33
mean age (years)	40 ± 8.4	47 ± 3.1
**sex**		
male	2	23
female	6	10
tumour size (cm)	11.8 (8–19)	12.0 (8.5–20)
AJCC stage III	8	33
**resection margin**		
marginal	4 (50%)	18 (55%)
wide	4 (50%)	15 (45%)
elapsed time from biopsy to tumour resection (days)	7.8 (5–11)	8.5 (5–14)
**site**		
proximal upper extremitiy	2	5
distal upper extremity	1	1
proximal lower extremitiy	5	24
distal lower extremity	0	3

**Table 3:** Combined therapy related complications

**Complication**	**preoperative group**	**postoperative group**	**total**
Wound healing disorder	2	3	5
Pathological fracture	0	2	2
Lymph edema	1	3	4

Wound healing disorders had to be treated by surgery in 25% in the preoperative radiation group and in 9% in the postoperative radiation group (p = 0.22, chi square). Pathological fractures following irradiation were only present in the postoperative group (6%). A lymph edema occurred in 12.5% (preoperative group) and 9% (postoperative group).

**Table 4:** Metastasis: incidence in preoperative versus postoperative irradiation group

	**metastasis**	
**radiotherapy**	No	yes

postoperative	17 (51.5%)	16 (48.5%)
preoperative	1 (12.5%)	7 (87.5%)
total	18 (43.9%)	23 (56.1%)

Metastatic disease was more likely to occur (p = 0.046) in the preoperative radiation therapy group. Most of the metastases (83%) occurred in multiple localisations almost simultaneously (n = 11) or in the lung (n = 8). Three times metastasis to lymph nodes (13%) and once to bone (4%) was noted.

**Table 5:** Clinical features of patients treated with preoperative radiotherapy

**Nr.**	**Age**	**Sex**	**Site**	**Size**	**Diag**	**Margin**	**FU/m**	**Met/m**	**Met/s**	**status**
27	35	F	2	13	3	marginal	10	5	Lung	DOD
81	60	M	1	8	1	marginal	11	5	Multi	DOD
203	60	F	4	8	1	Wide	59	46	Multi	DOD
206	48	F	4	13	5	Wide	13	6	Multi	DOD
331	77	F	1	19	3	Wide	6	2	Multi	DOD
342	26	M	3	12	1	marginal	65	8	Lung	NOD
355	54	F	4	10	1	Wide	14	12	multi	DOD

Average tumour size was 12 cm. Average follow-up was 25 months. Pat. 27 and 342 underwent thoracotomy for lung metastasis resection. Pat. 331 developed local recurrence one month after multiple metastases under palliative treatment.

**Table 6:** Clinical features of patients treated with postoperative radiotherapy

**Nr.**	**Age**	**Sex**	**Site**	**Size**	**Diag**	**Margin**	**FU/m**	**Met/m**	**Met/s**	**status**
4	31	M	4	9	3	Marginal	16	3	Lung	DOD
30	33	M	4	18	4	Wide	23	11	Multi	DOD
63	55	M	2	12	1	Wide	23	14	Lung	DOD
64	60	M	4	10	1	Wide	44	11	Lung	NOD
82	67	M	4	8.5	1	Marginal	15	3	Multi	DOD
137	61	M	1	10	1	Marginal	32	30	Lung	DOD
144	63	M	4	20	1	Wide	6	4	Lung	DOD
145	68	M	5	11.5	1	Marginal	81	64	Lymph	NOD
160	21	M	4	11	2	Marginal	31	21	Multi	DOD
165	31	F	2	15	2	Marginal	15	3	Multi	DOD
178	32	M	4	10	2	Wide	18	16	Multi	DOD
190	58	M	4	12	3	Marginal	24	12	Multi	DOD
198	54	F	1	13	2	Marginal	120	6	Lung	NOD
227	71	F	4	12	5	Marginal	39	38	Multi	DOD
239	27	M	2	9.5	5	Wide	33	31	Lymph	DOD
328	31	M	2	10	3	Marginal	8	4	Os	DOD

Average tumour size was 12 cm. Average follow-up was 33 months. Pat. Nr. 63, 64 and 198 underwent thoracotomy for lung metastasis resection. Pat. 64 developed local recurrence (treated with limb sparing surgery) 2 months prior to metastasis. Pat. 137 developed local recurrence after 6 months (treated with limb sparing surgery) and metastatic disease 24 months later. Pat. 190 developed local recurrence two months after metastatic disease occurred and palliative treatment had been initiated. Pat. 82, 145 and 239 were resected for regional lymph node metastasis. Pat. 82 and 239 developed progredient disease with further metastasis under palliative treatment.

**Abbreviations: **Sex: F = female, M = male site: 1 = upper extremity 2 = pelvis 3 = groin 4 = thigh 5 = popliteal fossa 6 = shank diagnosis: 1 = malignant fibrous histiocytoma 2 = leiomyosarcoma 3 = liposarcoma 4 = synovial sarcoma 5 = neurogenous sarcoma

FU/m: Follow-up/months

Met/m: Manifestation of metastatic disease/months after tumour resection

Met/s: Anatomical localisation of metastasis

status: DOD = died of disease NOD = no evidence of disease

**Table 7:** Local recurrence: preoperative versus postoperative therapy group

	**local recurrence**	
**radiotherapy**	no	Yes

postoperative	24 (70%)	9 (30%)
preoperative	7 (87.5%)	1 (12.5%)
total	31 (76%)	10 (24%)

There was no statistical significance (p = 0.38, chi square) in local control between the two examined groups. Local recurrence was treated with limb sparing surgery (n = 6), salvage amputation (n = 2) and palliatively in 2 cases of previous multiple metastasis.
